# Efficient Production of 2-Keto-l-Gulonic Acid via One-Step Fermentation Using *Gluconobacter oxydans* WTF0512 and *Ketogulonicigenium vulgare* WTF0114

**DOI:** 10.3390/microorganisms14050947

**Published:** 2026-04-22

**Authors:** Hongling Liu, Xiangxin Bu, Mingxia Jiao, Wenhu Chen, Xiangling Jiang, Haibo Yuan, Di Huang, Yi Jiang, Cheng Zhong, Tengfei Wang

**Affiliations:** 1 State Key Laboratory of Green Papermaking and Resource Recycling, Qilu University of Technology (Shandong Academy of Sciences), Jinan 250353, China; liuhongling@qlu.edu.cn (H.L.); amutou6@163.com (X.B.); 19853565610@163.com (M.J.); cwh0410@163.com (W.C.); 18053237309@163.com (X.J.); hbyuan@qlu.edu.cn (H.Y.); dihuang1992@qlu.edu.cn (D.H.); yijiang@qlu.edu.cn (Y.J.); 2 Shandong Provincial Key Laboratory of Biosensing and Microbial Intelligent Metabolic Regulation, School of Bioengineering, Qilu University of Technology (Shandong Academy of Sciences), Jinan 250353, China; 3 State Key Laboratory of Food Nutrition & Safety, College of Biotechnology, Tianjin University of Science and Technology, Tianjin 300457, China; czhong@tust.edu.cn; 4 Key Laboratory of Industrial Fermentation Microbiology (Ministry of Education), Tianjin University of Science and Technology, Tianjin 300457, China

**Keywords:** vitamin C, 2-keto-l-gulonic acid, *Gluconobacter oxydans*, *Ketogulonicigenium vulgare*, one-step fermentation

## Abstract

Currently, the main method for producing the vitamin C precursor 2-keto-l-gulonic acid (2-KLG) is a two-step fermentation process, in which secondary sterilization and fermentation processes result in higher costs and energy consumption. Consequently, the development of a one-step fermentation process is seen as a more desirable approach for 2-KLG production. In this study, we used *Gluconobacter oxydans* WTF0512 and *Ketogulonicigenium vulgare* WTF0114 as co-cultured strains for the production of 2-KLG from d-sorbitol via one-step fermentation. The fermentation behaviors of *G. oxydans* WTF0512 and *K. vulgare* WTF0114 were initially investigated. Subsequently, the fermentation process and medium were optimized, and the titer of 2-KLG reached 132.99 ± 0.52 g/L, with a molar conversion rate of 92.42%, which, to the best of our knowledge, is the highest production via one-step fermentation reported to date. These findings will provide a basis for developing a more economical large-scale one-step fermentation process for the production of 2-KLG.

## 1. Introduction

Vitamin C is a highly potent antioxidant and an indispensable nutrient for the human that has extensive industrial applications in the pharmaceutical, food, health, cosmetics, and aquaculture industries, with global requirements estimated at USD 1.9 billion by 2033 [1]. Presently, a three-bacterial two-step fermentation method, which was originally developed in the 1970s to produce the vitamin C precursor 2-keto-l-gulonic acid (2-KLG), has been adopted by almost all the main vitamin C producers [2]. In the two-step fermentation method, *Gluconobacter oxydans* is initially used to convert d-sorbitol to l-sorbose, which is followed by a co-culture system composed of *Ketogulonicigenium vulgare* (for the conversion of l-sorbose to 2-KLG) and the companion bacterium (to promote the growth and 2-KLG production of *K. vulgare*) that converts l-sorbose to 2-KLG [3]. Subsequently, 2-KLG is chemically converted to vitamin C [4]. Given that the two-step fermentation necessitates secondary sterilization and fermentation, there are certain drawbacks to this method of production, notably, large equipment investment, long fermentation periods, high labor costs, and high energy consumption [5]. Moreover, as a consequence of continuous research on traditional two-step fermentation methods, the process of vitamin C production is becoming increasingly more sophisticated, and market competition intensifies with each passing year. Consequently, to enhance productivity, reduce expenses, and upgrade product quality, there is a pressing need for more innovative approaches to the production processes. Accordingly, the development of a high-yielding one-step fermentation process, characterized by high conversion rates, short fermentation periods, and low costs, has become a particular focus of research in the field of vitamin C production.

Utilizing genetic engineering technology to modify microorganisms for the direct production of vitamin C via microbial fermentation can eliminate the chemical conversion of 2-KLG to vitamin C and simplify the production process. However, although the production of vitamin C via direct fermentation using genetically engineered bacteria has been realized, the titer is currently relatively low, and, consequently, it is difficult to meet the requirements of industrial production [6]. Nevertheless, significant progress has been made in the preparation of 2-KLG via one-step fermentation with subsequent chemical conversion of 2-KLG to vitamin C, and some studies have indicated the industrial production potential of the one-step fermentation method in terms of titer, fermentation period, and cost [7].

*G. oxydans* is not only characterized by simple nutritional requirements and vigorous growth but can also convert d-sorbitol to l-sorbose with high titer, conversion rates, and fermentation productivity [8]. Moreover, some *G. oxydans* strains have the capacity to produce 2-KLG from d-sorbitol. Therefore, relatively high-yielding production of 2-KLG via one-step fermentation can be realized by overexpressing genes associated with 2-KLG synthesis or heterologously expressing the dehydrogenases from *K. vulgare* that catalyze the conversion of l-sorbose to 2-KLG. In this regard, it has been demonstrated that using d-sorbitol as a precursor, *G. oxydans* T100 can directly produce 7.0 g/L 2-KLG. In addition, on the basis of the overexpression of l-sorbose dehydrogenase (SDH) and l-sorbosone dehydrogenase (SNDH) from *G. oxydans* T100 in *G. oxydans* G624, along with the further inhibition of the l-idonate pathway via chemical mutation and replacement of the original promoter with the *tufB* promoter from *Escherichia coli*, a 2-KLG titer of 130 g/L has been achieved from 150 g/L d-sorbitol, with a molar conversion rate of 79.70% [9]. Furthermore, by overexpressing selected SDH and SNDH fusion proteins with different linkers and PQQ gene cluster, a 2-KLG titer of 39.8 g/L has been obtained from 150 g/L d-sorbitol in *G. oxydans*/pGUC-*k0203*-GS-*k0095*-pqqABCDE [10]. Subsequently, by using scaffold proteins and enhancing the expression of PQQ in *G. oxydans*/pGUC-*tufB*-*SH3*-*sdh*-GGGGS-*sndh*-*tufB-SH3_lig_*-(GGGGS)_2_-*cutA*-*tufB*-*pqq*ABCDE, an increase in the production of 2-KLG to 42.6 g/L was obtained from 150 g/L d-sorbitol [11]. However, compared with the traditional two-step fermentation method, one-step fermentation generally has the disadvantages of low titer, the generation of by-products, and high costs [12]. This has accordingly provided an impetus to identify novel chassis microorganisms that could be utilized to facilitate the one-step production of 2-KLG. For example, Li et al. [13] have developed a comprehensive metabolic engineering strategy to establish and optimize a one-step 2-KLG fermentation process from d-sorbitol in *Pseudomonas putida* KT2440 and found that the recombinant strain was characterized by a significantly enhanced production of 2-KLG, with a titer of up to 6.5 g/L, representing a 15.48-fold increase compared to the original strain.

Utilizing traditional two-step fermentation strains and contracting fermentation from a two-step to a one-step process is highly desirable from both scientific research and practical perspectives. In this regard, *K. vulgare*, which is used to convert l-sorbose to 2-KLG in the second step of the two-step fermentation process, grows slowly and produces relatively low 2-KLG when cultured alone, and, consequently, requires the presence of a companion bacterium to promote its growth and 2-KLG production [14]. Wang et al. [15] have accordingly developed a two-bacteria one-step fermentation process using *K. vulgare* and *G. oxydans*, the 2-KLG titer can reached 59.1 g/L within 28 h. Moreover, following the simultaneous deletion of NADPH-dependent l-sorbose reductase B932-1330 and the PTS system transporter subunit IIA B932-1730 in *G. oxydans* H6, the titer of 2-KLG was increased to 76.56 g/L at 36 h.

However, although the production of 2-KLG via one-step fermentation using two strains has been achieved, given the relatively low titer, the production costs using this approach were higher than those of traditional two-step fermentation. Consequently, there is an imperative to enhance the titer of one-step fermentation processes for industrial production. In this study, the fermentation behaviors of *G. oxydans* WTF0512 and *K. vulgare* WTF0114 were preliminarily examined, with the primary focus on establishing a one-step fermentation system to achieve a high titer of 2-KLG from d-sorbitol. Through optimization of the fermentation process and medium to regulate the proportion and metabolic state of *G. oxydans* WTF0512 and *K. vulgare* WTF0114 in a one-step fermentation process, a high titer of 2-KLG was obtained. This study provides a promising strategy for developing an economically viable one-step fermentation process for industrial-scale 2-KLG production.

## 2. Materials and Methods

### 2.1. Strains and Culture Conditions

*G. oxydans* WTF0512 and *K. vulgare* WTF0114 were mutagenized, screened in our laboratory, and have been deposited in the China Center for Type Culture Collection (CCTCC NO: M20241208 and CCTCC NO: M2024406, respectively). *G. oxydans* WTF0512 was grown in seed medium containing 20 g/L d-sorbitol, 6 g/L yeast extract, and 1 g/L CaCO_3_. The seed medium for the monoculture of *K. vulgare* WTF0114 contained 20 g/L l-sorbose, 3 g/L corn-steep liquor (CSL), 10 g/L peptone, 3 g/L yeast extract, 3 g/L beef extract, 1 g/L urea, 1 g/L KH_2_PO_4_, 0.2 g/L MgSO_4_, and 1 g/L CaCO_3_. *G. oxydans* WTF0512 and *K. vulgare* WTF0114 were grown for 18 h and 28 h, respectively, under the following conditions: 250 mL flasks containing 50 mL of seed medium at 30 °C and 200 rpm.

For fermentation in a 5 L fermenter (Shanghai Bailun Biological Technology Co., Ltd., Shanghai, China), the basal fermentation medium contained defined d-sorbitol or l-sorbose, 10 g/L CSL, 12 g/L urea, 3 g/L yeast extract, 1 g/L KH_2_PO_4_, 0.2 g/L MgSO_4_, and 1 g/L CaCO_3_. The pH was automatically controlled by the addition of 20% (*w*/*v*) NaOH or 2 mol/L HCl. For a two-bacteria one-step fermentation, the initial fermentation took place at 30 °C. The agitation speed was maintained at 600 rpm, with an air flow rate of 1.5 vvm. The inoculum volume was 15%, with *G. oxydans* WTF0512 and *K. vulgare* WTF0114 in a defined ratio based on OD_600_. The monoculture of *G. oxydans* WTF0512 was cultivated in a basal fermentation medium containing 130 g/L of d-sorbitol, with an inoculation volume of 7.5% at 30 °C. The monoculture of *K. vulgare* WTF0114 was cultivated in a basal fermentation medium containing defined d-sorbitol and/or l-sorbose, with an inoculation volume of 7.5% at 30 °C.

### 2.2. Optimization of the One-Step Fermentation Process for 2-KLG Production

A 5 L fermenter with 70% liquid volume was used for fermentation experiments using a basal fermentation medium (Figure S1). Single-variable experiments were performed to investigate the effects of the OD ratio of *G. oxydans* WTF0512 to *K. vulgare* WTF0114 (1:1, 2:1, 4:1, 8:1, 16:1, and 32:1), inoculum volume (15%, 20%, 25%, and 30%), and phase control (pH and dissolved oxygen (DO)) on the fermentation process.

### 2.3. Optimization of the Fermentation Medium for a Two-Bacteria One-Step Fermentation

Single-variable experiments were performed to investigate the effects of the concentration of d-sorbitol (110 g/L, 120 g/L, 130 g/L, and 140 g/L), yeast extract (0, 1 g/L, 2 g/L, 3 g/L, and 4 g/L), CSL (6 g/L, 8 g/L, 10 g/L, and 12 g/L), and urea (8 g/L, 10 g/L, 12 g/L, 14 g/L, and 16 g/L) on the production of 2-KLG by *G. oxydans* WTF0512 and *K. vulgare* WTF0114. Fermentation was conducted at a temperature of 30 °C, with an inoculation volume of 25% (*v*/*v*) using *G. oxydans* WTF0512 and *K. vulgare* WTF0114 at an OD ratio of 4:1, a pH of 7.0, and phased DO control. During the early stage of fermentation, the agitation speed was 1000 rpm, with an airflow rate of 2.0 vvm. After the speed adjustment, the DO was maintained at 20% until the end of fermentation.

### 2.4. Analytical Methods

Cell density (OD_600_) was determined spectrophotometrically following the dissolution of CaCO_3_ in a 1% citric acid solution. The concentrations of 2-KLG, d-sorbitol, and l-sorbose were determined using an HPLC system (Shimadzu LC-20A; Kyoto, Japan) equipped with an Aminex HPX-87H column (Bio-Rad, Hercules, CA, USA) and a refractive index detector. The mobile phase was 3 mM H_2_SO_4_ flowing at a rate of 0.2 mL/min and 35 °C. The concentration of 2-keto-d-gluconic acid (2-KGA) and d-glucuronic acid were determined using the same HPLC system equipped with a Gemini NX-C18 column (5 μm, 250 × 4.6 mm) (Phenomenex, Torrance, CA, USA) and an ultraviolet detector at 205 nm and 35 °C. The mobile phase was sodium dodecyl sulfate (0.01 mol/L): acetonitrile (3%) = 2:3, with a flow rate of 0.2 mL/min.

Each fermentation experiment was independently performed in triplicate. Data are presented as the mean ± standard deviation. Statistical comparisons were conducted using one-way analysis of variance. Statistical analysis was performed using the Origin 9.4 software platform (OriginLab Corporation, Northampton, MA, USA).

## 3. Results and Discussion

### 3.1. Investigate the Growth and Fermentation Behaviors of G. oxydans WTF0512 in Monoculture

**Figure 1:**
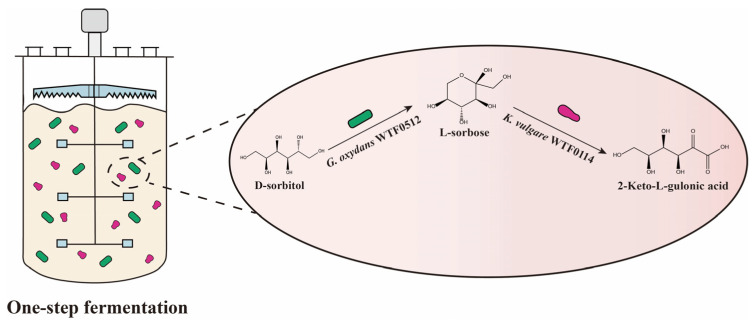
Schematic diagram of one-step fermentation.

**Figure 2:**
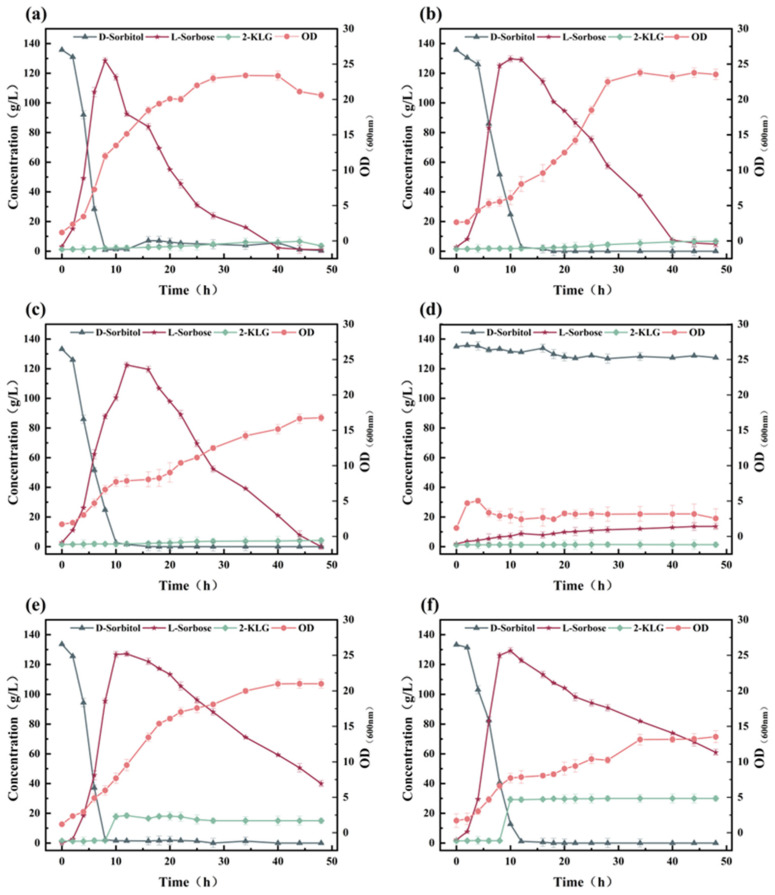
The influence of pH and 2-KLG concentration on growth and metabolism of *G. oxydans* WTF0512. (**a**–**d**) The effects on the growth and metabolism of *G. oxydans* WTF0512 when the pH values are 6.5, 7.0, 7.2, and 7.5, respectively. (**e**) The growth and metabolism of *G. oxydans* WTF0512 with the addition of 15 g/L 2-KLG after 9 h of fermentation at a pH of 7.0. (**f**) The growth and metabolism of *G. oxydans* WTF0512 with the addition of 30 g/L 2-KLG after 9 h of fermentation at a pH of 7.0.

To rationally design a one-step fermentation system for the 2-KLG production by *G. oxydans* WTF0512 and *K. vulgare* WTF0114 (Figure 1), the effects of pH and 2-KLG on the monoculture of *G. oxydans* WTF0512 in a 5 L fermenter were investigated. The findings indicate that under pH conditions ranging from 6.5 to 7.2, d-sorbitol rapidly converts to l-sorbose within 9–12 h. Subsequently, l-sorbose initiates degradation and is ultimately consumed (Figure 2a–c) [10,16]. As the pH value increases, the time for l-sorbose to reach its peak value lengthens, and the degradation rate of l-sorbose decelerates. Furthermore, the growth of *G. oxydans* WTF0512 is somewhat inhibited with an increase in pH value. At pH 7.5, the growth of *G. oxydans* WTF0512 and the conversion of d-sorbitol to l-sorbose is significantly inhibited, resulting in a maximum l-sorbose titer of only 13.66 g/L (Figure 2d). Additionally, a small amount of 2-KLG is observed (Figure 2a–d), in line with previous findings suggesting the existence of a full 2-KLG synthase pathway in *G. oxydans* [10].

To investigate the effect of 2-KLG on the degradation of l-sorbose by *G. oxydans* WTF0512, 15 g/L and 30 g/L 2-KLG were added when the l-sorbose reached its maximum value at 9 h. The results showed that, compared with the group without 2-KLG addition (Figure 2b), the degradation rate of l-sorbose was significantly decreased (Figure 2e,f). The residual l-sorbose in the group with 15 g/L 2-KLG was 51.20 g/L at 44 h (6.63 g/L under the group without 2-KLG addition), while the residual l-sorbose in the group with 30 g/L 2-KLG was 68.51 g/L at 44 h. Furthermore, a general decrease in biomass was also observed in both the group with 15 g/L 2-KLG (the maximum OD_600_ decreased from 23 to 21) and the group with 30 g/L 2-KLG (the maximum OD_600_ decreased from 23 to 14).

The results above indicate that *G. oxydans* WTF0512 has the ability to rapidly convert d-sorbitol into l-sorbose, with the rate of l-sorbose degradation being impacted by both pH and the concentration of 2-KLG. Therefore, precise control of fermentation conditions is essential when developing a two-bacteria one-step fermentation system for 2-KLG production.

### 3.2. Investigate the Fermentation Behaviors of K. vulgare WTF0114 in Monoculture

**Figure 3:**
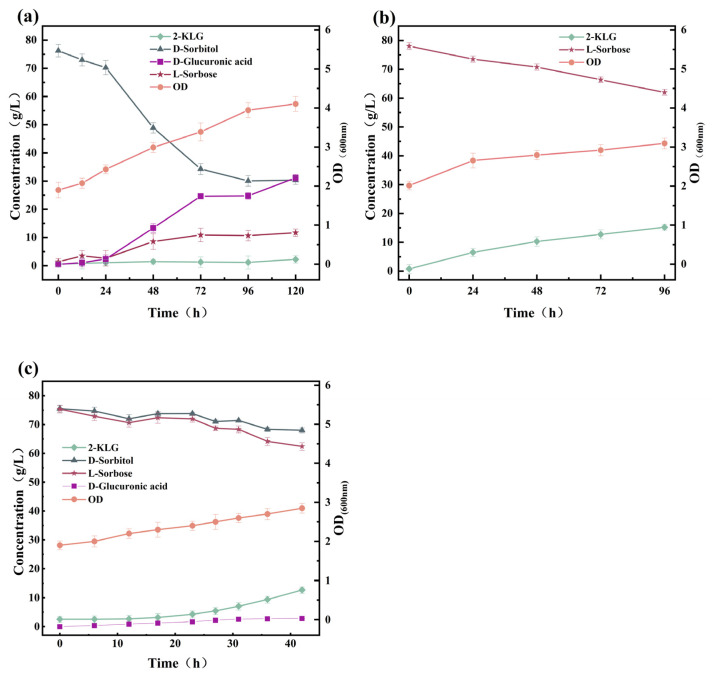
Effects of d-sorbitol and l-sorbose on the metabolism of *K. vulgare* WTF0114: (**a**) 75 g/L d-sorbitol; (**b**) 75 g/L l-sorbose; (**c**) 75 g/L d-sorbitol and 75 g/L l-sorbose.

In the traditional two-step fermentation process, *K. vulgare* can efficiently convert l-sorbose to 2-KLG by a co-culture system composed of the companion bacterium. Hence, the impact of d-sorbitol and l-sorbose on the metabolism of *K. vulgare* WTF0114 in monoculture was methodically examined in a 5 L fermenter. When *K. vulgare* WTF0114 was cultured in the presence of only d-sorbitol, the initial metabolic rate of d-sorbitol was slow within the first 24 h of fermentation. Subsequently, there was a notable acceleration in the metabolic activity of d-sorbitol, leading to the substantial generation of l-sorbose (11.70 g/L at 120 h) and the by-product d-glucuronic acid (31.19 g/L at 120 h) (Figure 3a). Moreover, 2.24 g/L of 2-KLG was observed at the end of fermentation and no by-product 2-KGA was generated. When *K. vulgare* WTF0114 was cultured in the presence of only l-sorbose, the biomass was relatively lower than when cultured with d-sorbitol alone. The l-sorbose was metabolized slowly, resulting in the production of 2-KLG only (15.5 g/L at 96 h), without the formation of the by-product d-glucuronic acid (Figure 3b). When *K. vulgare* WTF0114 was grown in a medium containing a mixture of d-sorbitol and l-sorbose (1:1, *w*/*w*), it metabolized both sugars simultaneously. l-sorbose was consumed at a higher rate compared to d-sorbitol, resulting in the production of 12.69 g/L of 2-KLG after 42 h (Figure 3c). Despite the existence of l-sorbose, *K. vulgare* WTF0114 still generates a small amount (2.85 g/L) of d-glucuronic acid as a by-product from d-sorbitol at 42 h. 2-KGA and d-glucuronic acid complicate downstream purification due to their structural similarity to 2-KLG, resulting in reduced separation efficiency, lower product purity, and increased processing costs [17,18]. Controlling these by-products is essential for optimizing the overall process economics.

The results above demonstrate that in the presence of d-sorbitol alone, *K. vulgare* WTF0114 exhibits a lag phase for d-sorbitol utilization during the initial 24 h of fermentation (Figure 3a), whereas *G. oxydans* WTF0512 could efficiently convert d-sorbitol to l-sorbose within 9–12 h of fermentation (Figure 2a–c). During the lag phase, *K. vulgare* WTF0114 provides *G. oxydans* WTF0512 with the necessary time to complete the transformation of d-sorbitol into l-sorbose, thereby preventing the production of d-glucuronic acid by *K. vulgare* WTF0114 using d-sorbitol. This observation highlights the potential for developing a two-bacteria one-step fermentation system using d-sorbitol as the primary substrate [19].

### 3.3. Optimization of the One-Step Fermentation Process

#### 3.3.1. Effects of *G. oxydans* WTF0512 to *K. vulgare* WTF0114 Ratio on Production of 2-KLG

**Figure 4:**
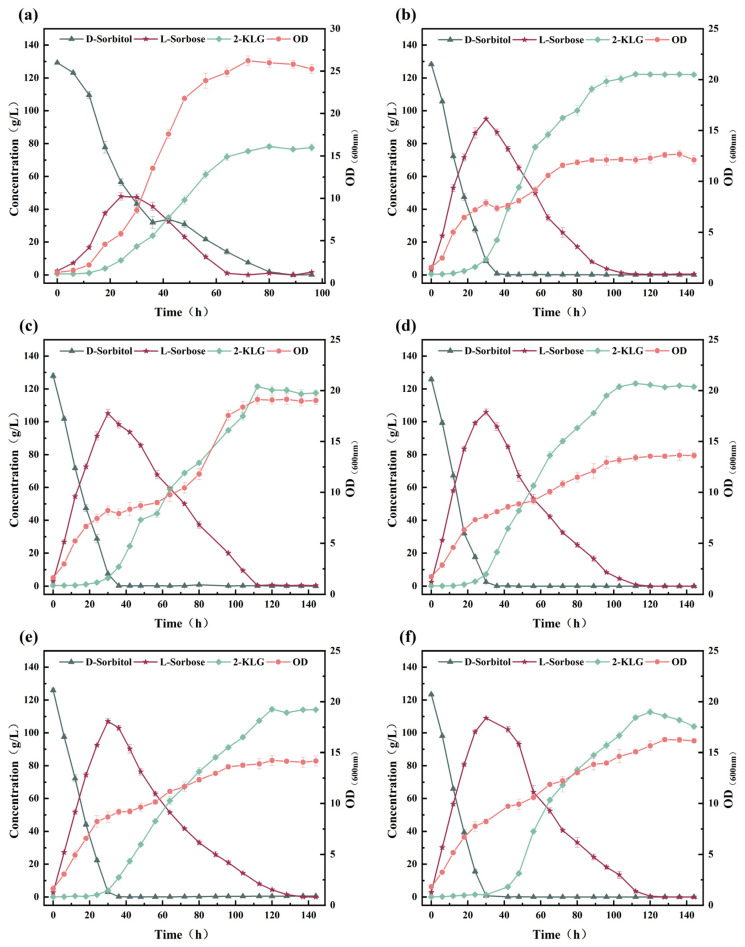
Effect of OD ratio of *G. oxydans* WTF0512 and *K. vulgare* WTF0114 on 2-KLG production by one-step fermentation. (**a**) 1:1; (**b**) 2:1; (**c**) 4:1; (**d**) 8:1; (**e**) 16:1; (**f**) 32:1.

Based on the fermentation behaviors of *G. oxydans* WTF0512 and *K. vulgare* WTF0114, a two-bacteria one-step fermentation system was developed using these two strains with 130 g/L of d-sorbitol. However, only 78.23 g/L of 2-KLG was obtained (Figure 4a). To assess the factors contributing to this inefficient production, we aimed to optimize the fermentation process by balancing the relationship between *G. oxydans* WTF0512 and *K. vulgare* WTF0114 via one-step fermentation to produce 2-KLG from d-sorbitol. The impact of the ratio of inoculated *G. oxydans* WTF0512 and *K. vulgare* WTF0114 on 2-KLG production was first investigated, as depicted in Figure 4. When the ratios of the OD of *G. oxydans* WTF0512 to *K. vulgare* WTF0114 were 2:1, 4:1, and 8:1, 122.31 ± 1.02, 121.51 ± 1.25, and 123.37 ± 1.34 g/L of 2-KLG were obtained at 112 h, respectively (Figure 4b–d). At excessively higher proportions of *G. oxydans* WTF0512 in the mixed seeds (OD ratios of 16:1 and 32:1) (Figure 4e,f), *K. vulgare* WTF0114 was at a competitive disadvantage in terms of nitrogen source acquisition during the early stages of fermentation. This resulted in a reduction in fermentation productivity and an extension of the fermentation period in the acid-producing stage (the conversion of l-sorbose to 2-KLG) during the latter stages of fermentation. Given the excessive growth of *G. oxydans* WTF0512 during the early stages of fermentation, this bacterium consumed larger amounts of l-sorbose, resulting in a reduction in the production of 2-KLG. In the one-step fermentation process, when the ratio of *G. oxydans* WTF0512 to *K. vulgare* WTF0114 ranged from 2:1 to 8:1, there was no significant difference in the production of 2-KLG, although production was highly robust and stable. Taking into account the potential changes in biological activities between strains with the same OD during the production process, we adopted a *G. oxydans* WTF0512 to *K. vulgare* WTF0114 OD ratio of 4:1 for subsequent investigations to enhance fermentation stability and productivity.

#### 3.3.2. Effects of Inoculum Volume on the Production of 2-KLG

**Figure 5:**
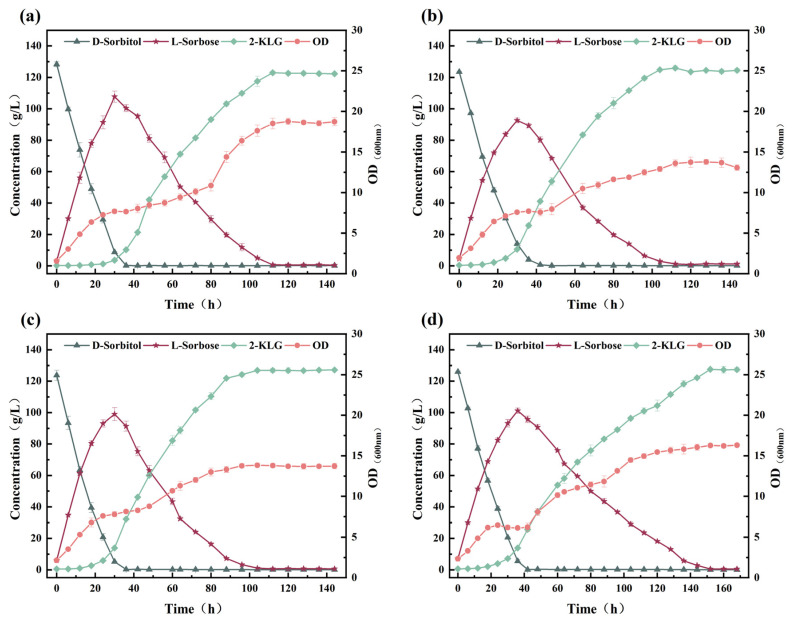
Effect of inoculum volume on 2-KLG production by one-step fermentation. (**a**) 15%; (**b**) 20%; (**c**) 25%; (**d**) 30%.

With respect to the effects of the volume of mixed-seed inoculum (*G. oxydans* WTF0512 and *K. vulgare* WTF0114) on 2-KLG production, we obtained a 2-KLG titer of 122.58 ± 0.91, 125.97 ± 1.32, and 126.94 ± 1.61 g/L at inoculum volumes of 15%, 20%, and 25%, respectively, with corresponding fermentation periods of 120, 112, and 104 h (Figure 5a–c). This marginal increase in 2-KLG production in response to an increase in the inoculum volume from 15% to 25% can be attributed to the fact that the mixed seeds contain a certain amount of l-sorbose, which contributes to an increased initial concentration of substrate. With a further increase in inoculation volume to 30%, there was an additional increase in the titer of 2-KLG to 127.55 ± 0.33 g/L, although this was accompanied by a marked extension in the fermentation period to 152 h (Figure 5d). Taking into consideration the 2-KLG titer and fermentation period, a 25% inoculum volume of mixed seeds was selected for subsequent studies.

The initial bacterial community structure and functional cooperation between *G. oxydans* WTF0512 and *K. vulgare* WTF0114 were jointly influenced by the inoculation ratio and inoculum volume. In a one-step system, *G. oxydans* WTF0512 plays a role beyond d-sorbitol oxidation. An optimal inoculum density of *G. oxydans* WTF0512 is likely to facilitate the growth of *K. vulgare* WTF0114 through mechanisms such as cellular turnover or metabolic secretion, including amino acids, vitamins, organic acids, and signaling molecules. The optimal inoculation condition strikes a delicate balance, enabling *G. oxydans* WTF0512 to facilitate rapid bioconversion and syntrophic interactions while maintaining the metabolic performance of *K. vulgare* WTF0114. Excessive and insufficient initial inoculum ratios or biomass levels of *G. oxydans* WTF0512 both have a negative impact on 2-KLG production efficiency.

#### 3.3.3. Effects of the Phased Control of pH and DO on the Production of 2-KLG

**Figure 6:**
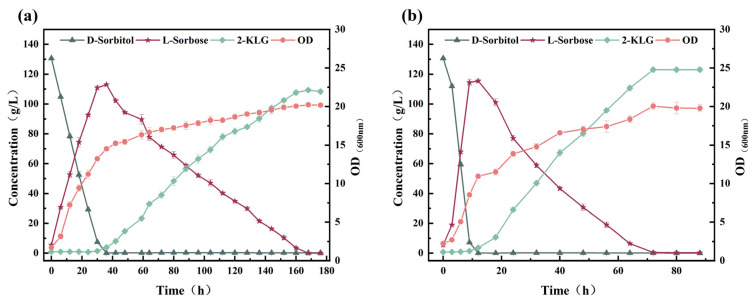
Effect of phased control of pH and DO on 2-KLG production by one-step fermentation. (**a**) Phased control of pH: with pH 6.5 during 0–24 h and kept at 7.0 from 24 h to the end of fermentation. (**b**) Phased control of DO: maintain 1000 rpm and 2.0 vvm from 0 to 12 h of fermentation, and adjust DO to 20% from 12 h until the end of fermentation.

Previous investigations have shown that *G. oxydans* WTF0512 and *K. vulgare* WTF0114 both grow better in slightly acidic environments, with the conversion of l-sorbose to 2-KLG being particularly favorable under neutral or weakly alkaline conditions [20,21]. The optimal pH for *K. vulgare* WTF0114 growth and 2-KLG production was found to be different from that of *G. oxydans* WTF0512 for the conversion of d-sorbitol to l-sorbose. In the one-step fermentation with *G. oxydans* WTF0512 and *K. vulgare* WTF0114, the synthesis of 2-KLG was found to be characterized by two discrete stages, in which *G. oxydans* WTF0512 initially contributes to a rapid conversion of d-sorbitol to l-sorbose during the early stage of fermentation, generating only a small amount of 2-KLG. Subsequently, when d-sorbitol was depleted, *K. vulgare* WTF0114 rapidly converts l-sorbose to 2-KLG. This two-phase phenomenon has also been reported in other studies [22]. Based on these findings, a phased pH control strategy was developed. The pH was maintained at 6.5 initially for the bioconversion of d-sorbitol to l-sorbose, and subsequently adjusted to 7.0 for the oxidation of l-sorbose to 2-KLG. This approach ensured a precise balance between the l-sorbose generation rate in the early stage and the optimal growth and 2-KLG production of *K. vulgare* WTF0114 in the later stage. However, this strategy did not significantly enhance d-sorbitol conversion to l-sorbose. Furthermore, it hindered the conversion of l-sorbose to 2-KLG as acidic conditions prompted *G. oxydans* WTF0512 to metabolize a portion of the l-sorbose. Consequently, the final 2-KLG titer reached only 109.25 ± 1.23 g/L after a fermentation period of 168 h (Figure 6a).

Growth kinetics from monocultures revealed that *G. oxydans* WTF0512 exhibits a significantly higher maximum specific growth rate (*μ*_max_ = 0.37/h, Figure 2) compared to *K. vulgare* WTF0114 (*μ*_max_ = 0.11/h, Figure 3). The profound distinction in growth kinetics implies that a high DO level would cause the strictly aerobic *G. oxydans* WTF0512 to overgrow. As a result, the excessive proliferation of *G. oxydans* WTF0512 is deliberately restrained by keeping a restricted DO level. Moreover, this optimized oxygen limitation inherently benefits the slower-growing *K. vulgare* WTF0114, which is physiologically better adapted to thrive in microaerobic environments [23]. Through staged control of DO, a yield of 123.86 ± 1.08 g/L of 2-KLG, with a molar conversion rate of 86.7%, was achieved at 72 h (Figure 6b).

Variations in DO and pH significantly influence the oxidative metabolic behavior of *G. oxydans* WTF0512 and *K. vulgare* WTF0114. Controlled modifications in DO levels significantly impact 2-KLG productivity, highlighting the importance of respiratory metabolism in regulating this fermentation process. Excessive oxygen availability can result in overoxidation of l-sorbose, impeding its efficient conversion into 2-KLG. Furthermore, elevated DO levels can increase the generation of reactive oxygen species (ROS) [24], posing a particular threat to *K. vulgare* due to its heightened susceptibility to oxidative stress. Conversely, pH modifications primarily impact enzymatic functions, maintenance of the proton motive force, and the equilibrium among various metabolic pathways and enzymes involved in 2-KLG formation [23]. Although pH regulation contributes to metabolic stability, its direct impact is typically less powerful than that of DO regulation. Hence, the dynamic control of oxygen levels is recognized as a more effective strategy for enhancing net 2-KLG accumulation in one-step fermentation processes.

In a previous two-bacteria one-step fermentation system using *K. vulgare* and *G. oxydans*, to reduce the competition between these two strains, Wang et al. [15] deleted genes involved in l-sorbose metabolism in *G. oxydans.* Similarly, to reduce competition between *G. oxydans* H24 and *K. vulgare* in a three-bacteria one-step fermentation process, a programmed cell death module based on the LuxI/LuxR quorum sensing (QS) system was established in *G. oxydans* H24. However, although effective to a certain extent, these strategies did not achieve a high titer and conversion rate of 2-KLG in the presence of high concentrations of d-sorbitol or l-sorbose. In this section, by optimizing fermentation conditions, including the inoculum ratio of *G. oxydans* WTF0512 to *K. vulgare* WTF0114, inoculum volume, and phased DO control, competition between *G. oxydans* WTF0512 and *K. vulgare* WTF0114 at a higher concentration of d-sorbitol (130 g/L) was mitigated. A yield of 123.86 ± 1.08 g/L of 2-KLG, with a molar conversion rate of 86.7%, was achieved at 72 h.

### 3.4. Optimization of the Fermentation Medium

**Figure 7:**
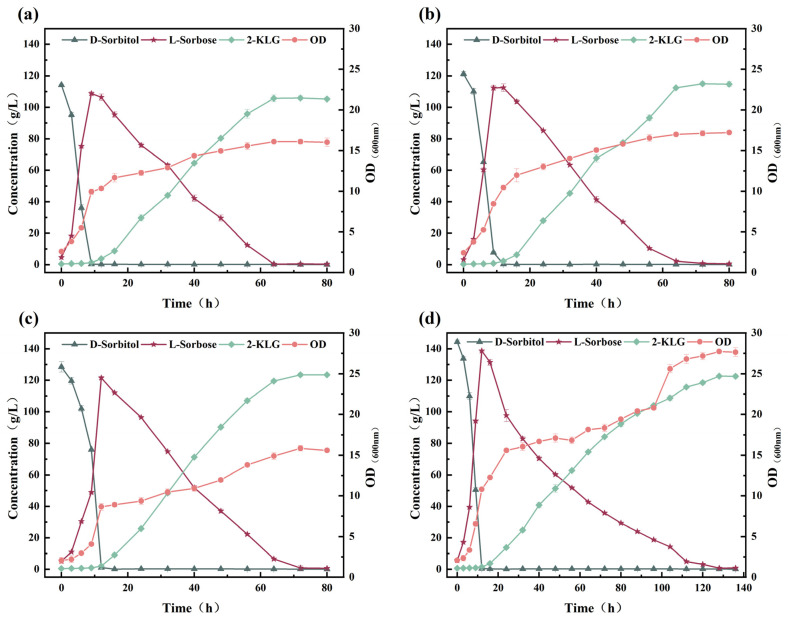
Effect of the concentration of d-sorbitol on 2-KLG production by one-step fermentation. (**a**) 110 g/L; (**b**) 120 g/L; (**c**) 130 g/L; (**d**) 140 g/L.

In the traditional two-step fermentation process for 2-KLG production, yeast extract has been established to be the optimal organic nitrogen source for *G. oxydans* [16], while CSL has been identified as the optimal organic nitrogen source for the second-stage fermentation of *K. vulgare* [25]. Urea serves as an inorganic nitrogen source for buffering pH fluctuations in the fermentation process. In this study, to further enhance 2-KLG production and shorten the fermentation period, we aimed to optimize the medium used for the one-step fermentation process. First, the impact of d-sorbitol concentration on the production of 2-KLG was investigated. In Figure 7, an increase in 2-KLG titer was observed when the initial d-sorbitol concentration was increased from 110 to 130 g/L. At concentrations of 110, 120, and 130 g/L, the fermentation periods were 64, 72, and 72 h, respectively, yielding 2-KLG titers of 105.70 ± 2.10, 114.97 ± 1.12, and 127.20 ± 1.04 g/L (Figure 7a–c). With the addition of 140 g/L of d-sorbitol, a 2-KLG titer of 122.63 ± 0.04 g/L was achieved, despite the fermentation period being extended to 128 h (Figure 7d). Furthermore, when the concentrations of d-sorbitol were between 110 and 140 g/L, *G. oxydans* WTF0512 required 9 to 12 h to completely convert d-sorbitol to l-sorbose, with a subsequent gradual conversion to 2-KLG. However, when the concentration of d-sorbitol exceeded 130 g/L, *K. vulgare* WTF0114 was inhibited by the corresponding high concentration of l-sorbose, as reflected in a prolongation of the time necessary to convert l-sorbose to 2-KLG, resulting in a prolonged fermentation time. Based on these findings, 130 g/L of d-sorbitol was selected for further study.

**Figure 8:**
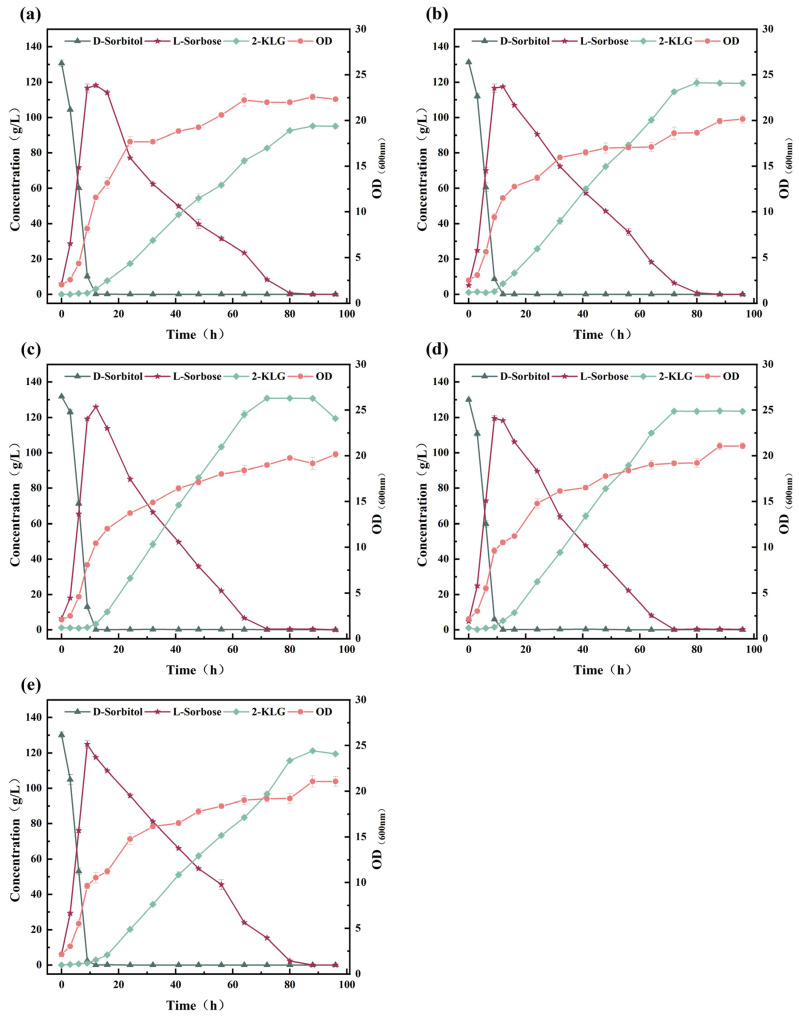
Effect of the concentration of yeast extract on 2-KLG production by one-step fermentation. (**a**) 0 g/L; (**b**) 1 g/L; (**c**) 2 g/L; (**d**) 3 g/L; (**e**) 4 g/L.

The effects of yeast extract concentration on the production of 2-KLG are shown in Figure 8. When the concentrations of yeast extract were 1, 2, 3, and 4 g/L, the titer of 2-KLG with 119.77 ± 2.31, 130.82 ± 1.25, 123.52 ± 0.21, and 121.20 ± 0.22 g/L was obtained, respectively, with corresponding fermentation periods of 88, 72, 72, and 88 h (Figure 8b–e). Comparatively, in the absence of yeast extract in the medium, a 2-KLG titer of only 95.12 ± 0.82 g/L was obtained, with a fermentation period of 88 h (Figure 8a). In addition, we found that at yeast extract concentrations between 2 and 4 g/L, the concentration of l-sorbose in the one-step fermentation system was slightly higher than that in the absence of yeast extract or when provided at 1 g/L. These findings indicate that yeast extract enhances the rate at which d-sorbitol is converted to l-sorbose by *G. oxydans* WTF0512. Notably, the concentration of yeast extract in the fermentation medium not only influenced the conversion rate of *G. oxydans* WTF0512 to convert d-sorbitol to l-sorbose, but also the conversion of l-sorbose into 2-KLG mediated by *K. vulgare* WTF0114. When the concentration of yeast extract in the medium was insufficient (0 or 1 g/L), there was a slight decrease in the rate at which d-sorbitol was converted to l-sorbose. It is predictable that under such conditions, the rate at which *K. vulgare* WTF0114 produces 2-KLG may not be rapid enough to suppress the metabolic consumption of l-sorbose by *G. oxydans* WTF0512 during the 2-KLG production stage [22], given the lack of nutrients [26]. Conversely, when the concentration of yeast extract was excessively high, the molar conversion rate of d-sorbitol to 2-KLG decreased by 4.98% to 87.41%, which can be attributed to the fact that *G. oxydans* WTF0512 consumes larger quantities of l-sorbose during the 2-KLG production stage. Upon considering these results, we determined that a yeast extract concentration of 2 g/L is optimal for the one-step fermentation process.

**Figure 9:**
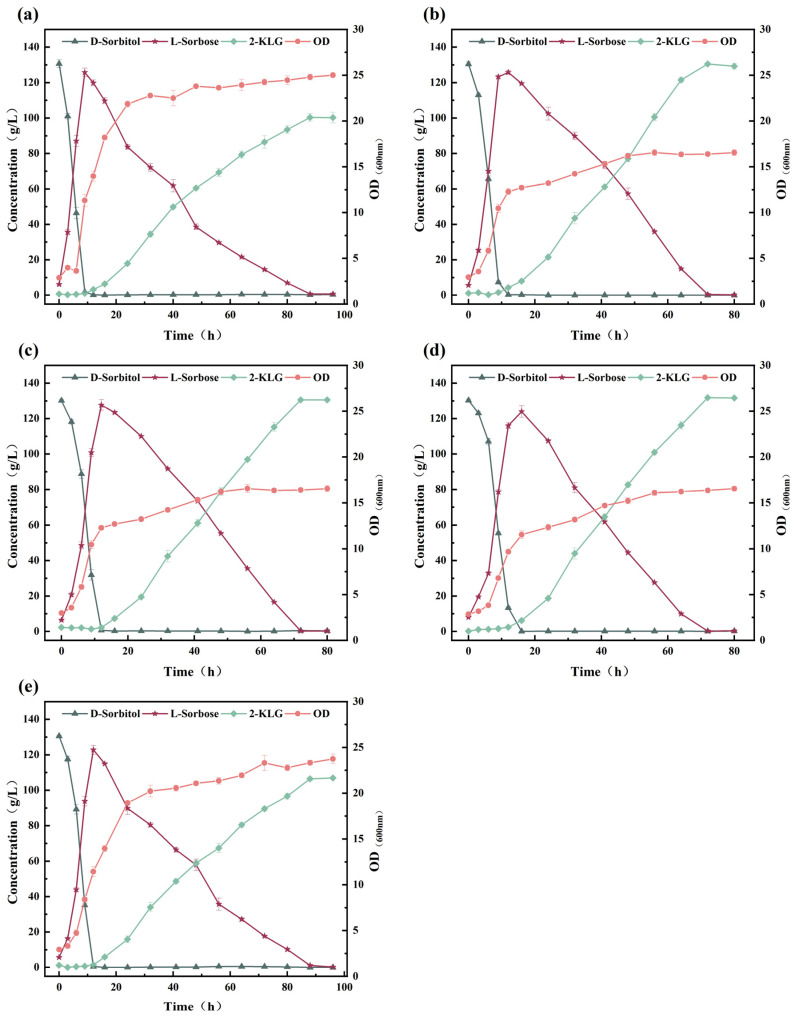
Effect of the concentration of urea on 2-KLG production by one-step fermentation. (**a**) 8 g/L; (**b**) 10 g/L; (**c**) 12 g/L; (**d**) 14 g/L; (**e**) 16 g/L.

Urea, in the second step of the conventional three-bacteria two-step fermentation process for 2-KLG production, serves not only as a reserve base for pH stabilization but also as an inorganic nitrogen source [27]. Our single-factor experiments revealed that 2-KLG titers of 100.34 ± 2.15, 131.48 ± 0.65, 130.98 ± 0.65, 131.82 ± 0.59, and 106.43 ± 1.02 g/L were obtained using medium supplemented with 8, 10, 12, 14, and 16 g/L urea, respectively (Figure 9a–e). Comparable titers of 2-KLG were achieved with urea concentrations of 10, 12, and 14 g/L over a 72 h fermentation period (Figure 9b–d). Taking into consideration the production costs, 10 g/L urea was selected for the one-step fermentation process.

**Figure 10:**
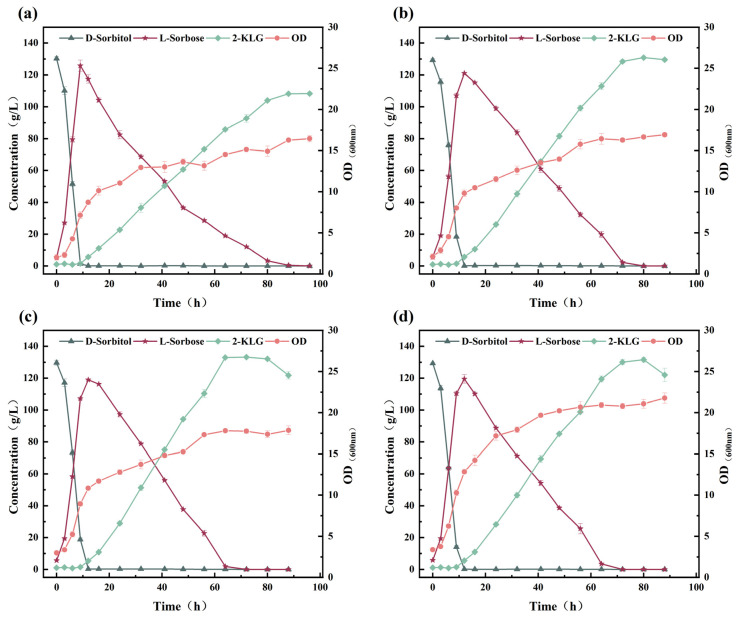
Effect of the concentration of CSL on 2-KLG production by one-step fermentation. (**a**) 6 g/L; (**b**) 8 g/L; (**c**) 10 g/L; (**d**) 12 g/L.

With respect to the concentration of CSL, we obtained 2-KLG titers of 108.14 ± 0.47, 130.87 ± 0.58, 132.99 ± 0.52, and 130.03 ± 1.62 g/L using medium supplanted with 6, 8, 10, and 12 g/L CSL, respectively, with corresponding fermentation periods of 88, 80, 64, and 72 h (Figure 10a-d). Given that 10 g/L CSL resulted in the highest 2-KLG titer and shortest fermentation period, it was selected as the optimal concentration (Figure 10c). When cultured in a medium supplemented with 6 g/L CSL, the growth of *K. vulgare* WTF0114 was restricted due to an insufficient nitrogen source, resulting in a prolonged fermentation period and a lower titer (Figure 10a). Conversely, at a CSL concentration of 12 g/L (Figure 10d), the excessive growth of *K. vulgare* led to rapid degradation of 2-KLG [12]. The influence of nitrogen sources on the production of 2-KLG is not only limited to providing nutrients, but may also involve metabolic regulation in the co-culture system of *G. oxydans* WTF0512 and *K. vulgare* WTF0114. Complex nitrogen sources, such as yeast extract and CSL, supply amino acids, vitamins, and nucleotides to potentially fulfill the auxotrophic needs of *K. vulgare* WTF0114, thereby supporting its metabolic functions throughout fermentation.

In conclusion, the optimized fermentation medium in this study consisted of 130 g/L d-sorbitol, 2 g/L yeast extract, 10 g/L CSL, and 10 g/L urea. Using this medium, the titer of 2-KLG reached 132.99 ± 0.52 g/L at 64 h (Figure 10d), with a molar conversion rate of 92.42% (taking the l-sorbose in the seed culture into account). Additionally, no by-products (2-KGA and d-glucuronic acid) were detected in the fermentation broth (Figures S2 and S3). The key fermentation metrics and parameters obtained during the optimization process are comprehensively summarized and compared in Table 1.

## 4. Conclusions

In this study, high 2-KLG production was achieved in a one-step fermentation process by utilizing a co-culture system of *G. oxydans* WTF0512 and *K. vulgare* WTF0114. Through the optimization of the fermentation process and culture medium composition, considering the distinct growth and fermentation characteristics of these two strains, a 2-KLG titer of 132.99 ± 0.52 g/L was achieved within 64 h of fermentation, with a molar conversion rate of 92.42%. To the best of our knowledge, the titer and molar conversion rate are the highest to date for 2-KLG production via two-bacteria one-step fermentation [9,15,28,29]. Genetic engineering techniques could further enhance *G. oxydans*’s capacity to produce and secrete nutrients that boost *K. vulgare* growth and 2-KLG production. Additionally, by blocking specific pathways in *K. vulgare* related to the metabolic consumption of l-sorbose or d-sorbitol, it is possible to enhance the titer and conversion rate of 2-KLG in the presence of high d-sorbitol concentrations, thus reducing the period of a one-step fermentation process.

## Figures and Tables

**Table 1:** The impact of crucial fermentation conditions on the production of 2-KLG in a one-step fermentation process.

Factors	Value	2-KLG Yield (g/L)	Time (h)	Volumetric Productivity (g/L/h)	Molar Conversion Rate (%)
Inoculation ratio of *G. oxydans* WTF0512 to *K. vulgare* WTF0114	1:1	78.23 ± 1.02	80	0.978	54.37
	2:1	122.31 ± 1.02	112	1.092	85.00
	4:1	121.51 ± 1.25	112	1.085	84.45
	8:1	123.37 ± 1.34	112	1.102	85.74
	16:1	114.44 ± 1.43	120	0.954	79.53
	32:1	112.76 ± 1.89	120	0.940	78.36
Inoculum volume (*v*/*v*)	15%	122.58 ± 0.91	112	1.094	85.19
	20%	125.97 ± 1.32	112	1.125	87.54
	25%	126.94 ± 1.61	104	1.221	88.22
	30%	127.55 ± 0.33	152	0.839	88.64
Phased control of pH and DO	Phased control of pH	109.25 ± 1.23	168	0.650	75.93
	Phased control of DO	123.86 ± 1.08	72	1.720	86.08
d-sorbitol concentration (g/L) *	110	105.70 ± 2.10	64	1.652	86.23
	120	114.97 ± 1.12	72	1.597	86.29
	130	127.20 ± 1.04	72	1.767	88.40
	140	122.63 ± 0.04	128	0.958	79.35
Yeast extract concentration (g/L)	0	95.12 ± 0.82	88	1.081	66.10
	1	119.77 ± 2.31	88	1.361	83.24
	2	130.82 ± 1.25	72	1.817	90.92
	3	123.52 ± 0.21	72	1.716	85.84
	4	121.20 ± 0.22	88	1.377	84.23
Urea concentration (g/L)	8	100.34 ± 2.15	88	1.140	69.73
	10	131.48 ± 0.65	72	1.826	91.37
	12	130.98 ± 0.65	72	1.819	91.03
	14	131.82 ± 0.59	72	1.831	91.62
	16	106.43 ± 1.02	88	1.209	73.96
CSL concentration (g/L)	6	108.14 ± 0.47	88	1.229	75.15
	8	130.87 ± 0.58	80	1.636	90.75
	10	132.99 ± 0.52	64	2.078	92.42
	12	130.03 ± 1.62	72	1.806	90.37

* The substrate transported from the seed culture in the ultimate fermentation broth totaled 5 g/L (computed according to the final volume) and was excluded from the variable parameters being investigated.

## Data Availability

Data are contained within the article and the Supplementary Materials. Further inquiries can be directed to the corresponding author.

## Supplementary Materials

The following supporting information can be downloaded at https://www.mdpi.com/article/10.3390/microorganisms14050947/s1, Figure S1: The schematic representation of the 5 L fermenter employed in the two-bacteria one-step fermentation process for the production of 2-KLG. Each fermenter is equipped with individual temperature, pH, and dissolved oxygen control systems. Figure S2: (A) HPLC analysis of the 2-KLG and by-products using Gemini NX-C18 column. (B) HPLC analysis of the d-sorbitol, l-sorbose, and 2-KLG using Aminex HPX-87H column. a, the mixed standard samples of 2-KLG, 2-KGA, and d-glucuronic acid; b, the fermentation sample collected at 64 h; c, the mixed standard sample of 2-KLG, d-sorbitol, and l-sorbose; d, the fermentation sample collected at 12 h; e, the fermentation sample collected at 64 h. Figure S3: The HPLC results illustrate the variations in d-sorbitol, l-sorbose, and 2-KLG concentrations over the course of fermentation. Purple, d-sorbitol; Red, l-sorbose; Green, 2-KLG.
