# Supplementary material for: Efficient Production of 2-Keto-l-Gulonic Acid via One-Step Fermentation Using Gluconobacter oxydans WTF0512 and Ketogulonicigenium vulgare WTF0114
Source: Microorganisms. 2026 Apr 22;14(5):947. doi: 10.3390/microorganisms14050947 (PMC13209538; doi:10.3390/microorganisms14050947)
Supplement: Supplementary file 1 [file microorganisms-14-00947-s001.zip › microorganisms-4252552-supplementary.pdf]

Electronic Supplementary Information

Efficient Production of 2-Keto-L-Gulonic Acid via One-Step Fermentation Using *Gluconobacter oxydans* WTF0512 and *Ketogulonicigenium vulgare* WTF0114

Hongling Liu [1,2], Xiangxin Bu [1,2], Mingxia Jiao [1,2], Wenhu Chen [1,2], Xiangling Jiang [1,2], Haibo Yuan [1,2], Di Huang [1,2], Yi Jiang [1,2], Cheng Zhong [3,4] and Tengfei Wang [1,2,*]

[1] State Key Laboratory of Green Papermaking and Resource Recycling, Qilu University of Technology (Shandong Academy of Sciences), Jinan 250353, China; liuhongling@qlu.edu.cn (H.L.); amutou6@163.com (X.B.); 19853565610@163.com (M.J.); cwh0410@163.com (W.C.); 18053237309@163.com (X.J.); hbyuan@qlu.edu.cn (H.Y.); dihuang1992@qlu.edu.cn (D.H.); yijiang@qlu.edu.cn (Y.J.)

[2] Shandong Provincial Key Laboratory of Biosensing and Microbial Intelligent Metabolic Regulation, School of Bioengineering, Qilu University of Technology (Shandong Academy of Sciences), Jinan 250353, China

[3] State Key Laboratory of Food Nutrition & Safety, College of Biotechnology, Tianjin University of Science and Technology, Tianjin 300457, China; czhong@tust.edu.cn

[4] Key Laboratory of Industrial Fermentation Microbiology (Ministry of Education), Tianjin University of Science and Technology, Tianjin 300457, China

* Correspondence: wangtengfeisci@163.com; Tel./Fax: +86-0531-8963-1901

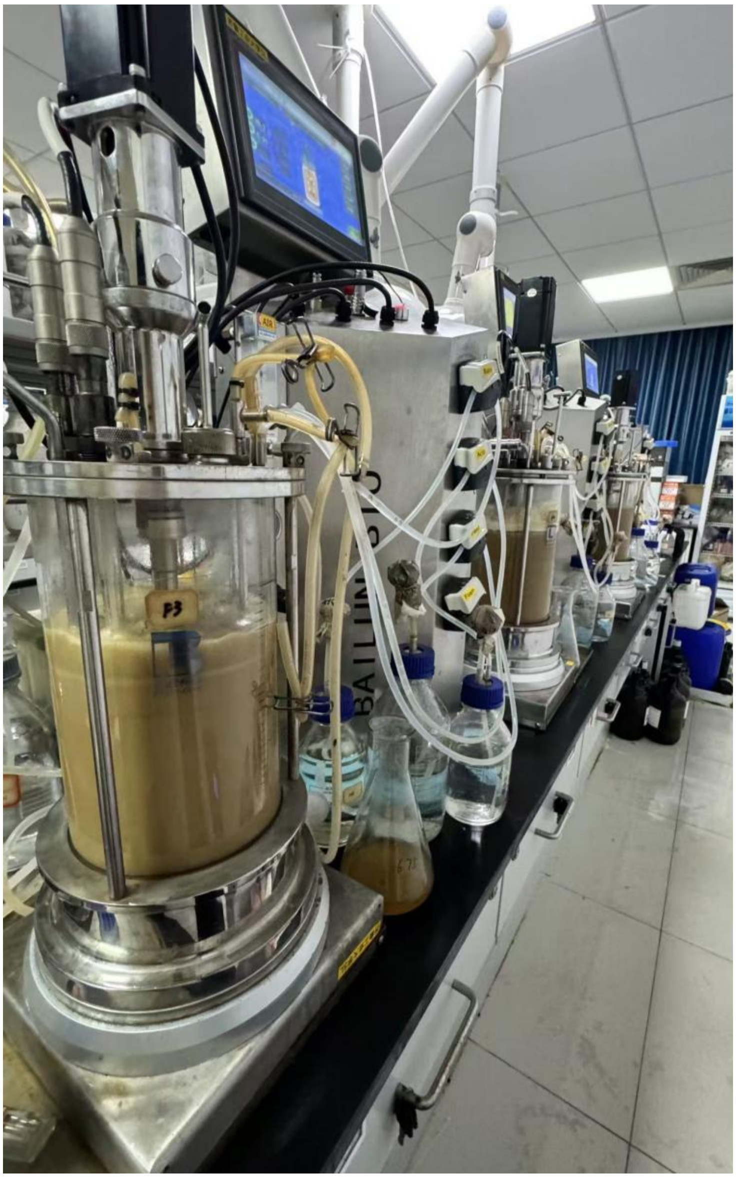

Figure S1. The schematic representation of the 5-L fermenter employed in the two-bacterial one-step fermentation process for the production of 2-KLG. Each fermenter is equipped with individual temperature, pH, and dissolved oxygen control systems.

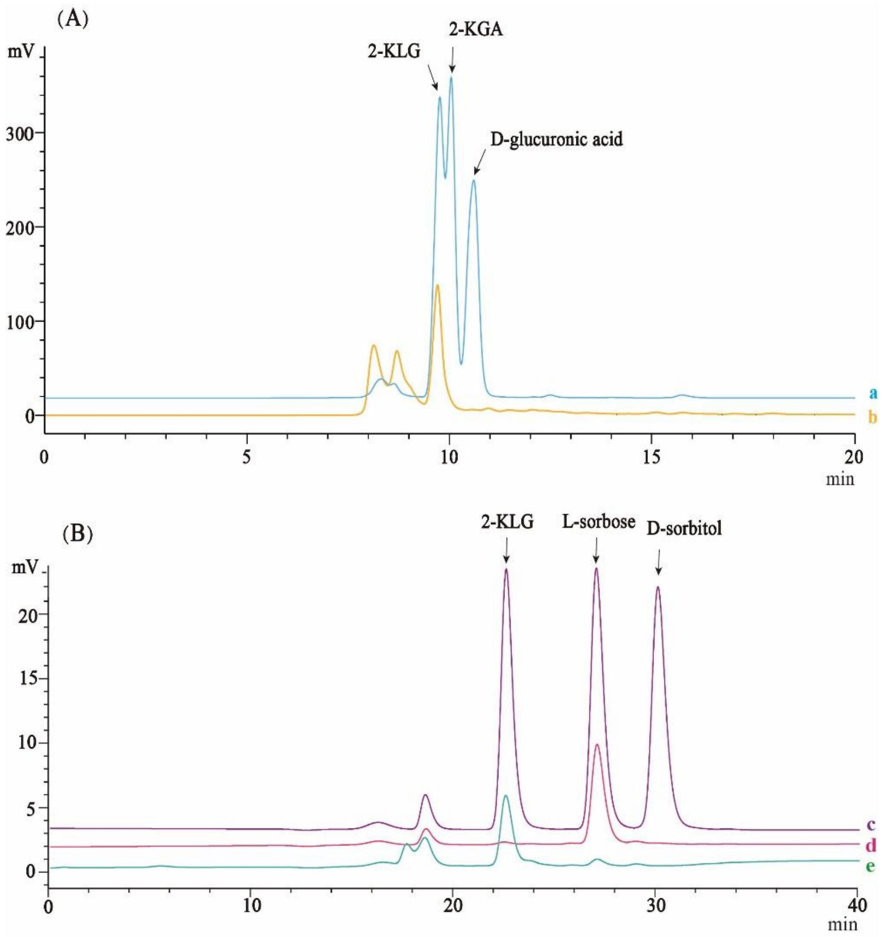

Figure S2. (A) HPLC analysis of the 2-KLG and by-products using Gemini NX-C18 column. (B) HPLC analysis of the D-sorbitol, L-sorbose, and 2-KLG using Aminex HPX-87H column. a, the mixed standard samples of 2-KLG, 2-KGA, and D-glucuronic acid; b, the fermentation sample collected at 64 h; c, the mixed standard sample of 2-KLG, D-sorbitol, and L-sorbose; d, the fermentation sample collected at 12 h; e, the fermentation sample collected at 64 h.

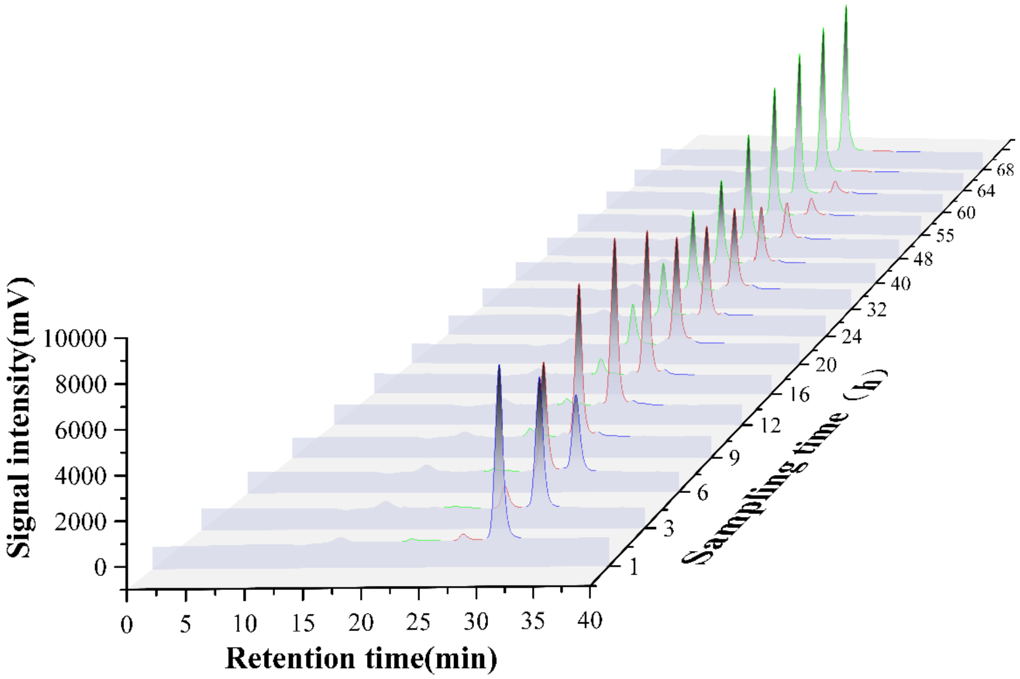

Figure S3. The HPLC results illustrate the variations in D-sorbitol, L-sorbose, and 2-KLG concentrations over the course of fermentation. Purple, D-sorbitol; Red, L-sorbose; Green, 2-KLG.
